# Maternal uniparental disomy of chromosome 7: how chromosome 7-encoded imprinted genes contribute to the Silver–Russell phenotype

**DOI:** 10.1186/s13148-025-01867-3

**Published:** 2025-04-30

**Authors:** Matthias Begemann, Anna Lengyel, Eva Pinti, Árpád Ferenc Kovács, György Fekete, Svea Stratmann, Jeremias Krause, Miriam Elbracht, Florian Kraft, Thomas Eggermann

**Affiliations:** 1 Medical Faculty, Centre for Human Genetics and Genome Medicine, RWTH University Aachen, Pauwelsstr. 30, 52074 Aachen, Germany; 2 Pediatric Center Tuzolto Street Department, Semmelweis University, Budapest, Hungary; 3 Science for Life Laboratory, Department of Immunology, Genetics, and Pathology, Uppsala University, Uppsala, Sweden

**Keywords:** Silver–Russell syndrome, Maternal uniparental disomy of chromosome 7, GRB10, MEST, Deletion

## Abstract

**Background:**

Silver–Russell syndrome (SRS) is a rare congenital growth disorder which is associated with molecular alterations affecting imprinted regions on chromosome 11p15 and maternal uniparental disomy of chromosome 7 (upd(7)mat). In 11p15, imprinted regions contributing to the SRS phenotype could be identified, whereas on chromosome 7 at least two regions in 7q32 and 7p13 are in discussion as SRS candidate regions. We report on DNA and RNA data from upd(7)mat patients and a monozygotic twin pair with a postnatal SRS phenotype carrying a small intragenic deletion within *GRB10* to delineate the contribution of upd(7)mat and imprinted genes on this chromosome to the SRS phenotype.

**Results:**

Genome sequencing in the monozygotic twins revealed a 18 kb deletion within the paternal allele of the *GRB10* gene. Expression of *GRB10* in blood of the twins as well as in cells from upd(7)mat and upd(7q)mat patients was not altered, whereas RNAseq indicates noticeable changes of the expression of other genes encoded by chromosomes 7 and other genomic regions.

**Conclusions:**

Our data indicate that intrauterine growth restriction as the prenatal phenotype of upd(7)mat is caused by defective paternal alleles of the 7q32 region, as well as by overexpression of the maternal *GRB10* allele whereas a defective *GRB10* paternal allele does not cause this feature. The altered expression of *MEST* in 7q32 by upd(7)mat is associated with the complete SRS phenotype, whereas maternalization or deletion of the paternal *GRB10* copy and duplication of the chromosomal region 7p12 are associated with a postnatal SRS-like phenotype.

**Supplementary Information:**

The online version contains supplementary material available at 10.1186/s13148-025-01867-3.

## Background

Silver–Russell syndrome (SRS) is a congenital syndrome characterized by severe intrauterine and postnatal growth restriction (IUGR, PNGR), relative macrocephaly with a characteristic facial gestalt, feeding difficulties, asymmetry of body and length and further less constant features [1]. Molecularly, it belongs to the group of imprinting disorders (ImpDis) as disturbances of chromosomal regions regulated by genomic imprinting can be identified in the majority of patients. However, SRS is unique among ImpDis as it is associated at least with two different chromosomes harbouring differentially methylated regions (DMRs). Among molecularly diagnosed SRS cases, more than 67% show a loss of methylation at the imprinting control region 1 in 11p15 (IC1 LOM), but additionally more than 15% exhibit a maternal uniparental disomy of chromosome 7 (upd(7)mat) [2]. However, the clinical heterogeneity of SRS and the nonspecificity of its key features results in an overlap with other growth retardation disorders, in particular with Temple syndrome (TS14). TS14 is another imprinting disorder associated with alterations of the *MEG3*:alt-TSS DMR in 14q32 and is detectable in more than 8% of patients referred for genetic SRS testing [2].

Both molecular subgroups are associated with similar phenotypes, but patients with IC1 LOM exhibit a lower birth length and weight and more additional congenital anomalies (e.g. protruding forehead, relative macrocephaly) than those with upd(7)mat. Body asymmetry is less frequent in upd(7)mat as is not associated with molecular mosaicism; in contrast, IC1 LOM commonly occurs as mosaic and might therefore result in hemihypotrophy. Finally, cognitive impairment (global developmental delay, verbal dyspraxia, learning difficulties) is a characteristic feature in many upd(7)mat patients [3].

In fact, upd(7)mat was the first genetic constitution consistently described in patients with SRS nearly 30 years ago [4], but its functional consequence is still unknown. Uniparental disomy (UPD) in general is the result of a chromosomal nondisjunction, and it can affect the clinical outcome by three mechanisms:

(a) By a hidden or undetected trisomy. The majority of upd(7)mat cases is caused by a maternal meiotic error and a trisomic zygote, followed by a rescue and loss of the paternal chromosome [5]. Depending on the time of formation, upd(7)mat can be associated with trisomy 7 mosaicism, and single cases with trisomy 7 mosaicism in extraembryonic cells have been reported (for review: [5]). However, trisomy 7 mosaicism has not yet been reported in SRS, though it should be noted that diagnostic testing in SRS is commonly based on lymphocyte DNA, and therefore, trisomy 7 might escape detection. Furthermore, in case (hidden) trisomy 7 mosaicism would mimic a upd(7)mat phenotype, the same clinical features should occur in patients with paternal uniparental disomy of chromosome 7 patients (upd(7)pat), but this is not the case [6].

(b) By homozygosity of a recessive pathogenic variant in an isodisomic UPD region causing a monogenetic disorder. Nevertheless, there is no common isodisomic region in patients with upd(7)mat which excludes homozygosity of an autosomal recessive variant or gene to cause SRS features [5].

(c) By disturbance of the balanced and parent-of-origin specific monoallelic expression of genomically imprinted genes. The observations that chromosome 7 harbours at least three DMRs, and that upd(7)mat is associated with a specific phenotype (i.e. SRS) whereas paternal upd(7) is not [7], indicates that imprinted genes play a role in the aetiology of the disease.

Up to now, three genomically imprinted domains of putative clinical relevance have been identified on chromosome 7, i.e. *MEST*:alt-TSS DMR in 7q32 (for review: [8]), *PEG10*:TSS DMR in 7q21.3 and *GRB*10:alt-TSS DMR in 7p12.1, but further DMRs are in discussion [9].

Among these three regions, the 7q32.2 DMR has been regarded as an obvious candidate region for SRS, as cases with maternal UPD restricted to the tip of the long arm (so-called segmental upd(7q)mat) exhibit the upd(7)mat phenotype [10] (Table 1). Furthermore, patients with deletions of *MEST*:alt-TSS DMR and the *MEST* gene affecting the paternal allele also show characteristics of SRS (Table 1). However, pathogenic variants in genes underlying the control of the *MEST*:alt-TSS DMR genes (i.e. the paternally expressed *MEST*, its imprinted antisense RNA *MESTIT1* [11], *COPG2* and its antisense transcript *CIT1*) have not yet been reported despite their coding region has been addressed in next-generation sequencing studies in patients with SRS features [12]. The physiological function of MEST in currently unknown, but *Mest* knock-out mice showed a reduced body weight and fat mass. Additionally, an impact on social and maternal behaviour is currently in discussion (for review: [13]).

**Table 1:** Comparison of the major clinical findings of SRS (based on the NH scoring system(41)) in our patients with the available data from the literature about patients with *GRB10* and *MEST* variants, as well as upd(7)mat/upd(7q)mat

	7p12							7q32			upd(7)mat					11p15
	Twin 1 M38099	Twin 2 M38100	*GRB10* GOM	6.1 Mb deletion of paternal allele	21.9 Mb deletion of paternal allele	Del *GRB10* exon 4–5 of maternal allele	7p12 Dup	upd(7q)mat	*MEST* deletion of the paternal allele		Wakeling et al	Azzi et al	Vimercati et al	Fuke et al	unpublished	IC1 LOM
Number of patients			1	1	1	1	3	5	1	1	20	11	17	10	34	29
Reference	Present study		(27)	(38)	(39)	(42)	(24, 25)	(10, 43)	(44)	(45)	(1)	(41)	(46)	(47)	Own data	(41)
*Clinical Features:*																
SGA	No	No	Yes**	No	No	NA	1/3	2/5	− 1.58 SD	− 3.0 SD	70%	72.7%	82%	100%	79.2%	100%
PNGR	(No)***	Yes	Yes**	No	No	NA	3/3	5/5	− 1.98 SD	− 1.67 SD	65%	90.9%	88%	100%	95.8%	100%
Relative Macrocephaly	Yes	Yes	Yes	Yes	No	NA	0/2	3(+ 1)/5	Yes	No	90%	81.8%	87%	100%	42.9%	96.9%
Protruding forehead	Yes	Yes	Yes	No	No	NA	1/3	2/4	Yes	Yes	60%	100%	70%	70%	90.9%	97%
Body asymmetry	No	No	No	No	No	NA	0/3	0/5	No	No	10%	27.3%	35%	30%	9.1%	94,3%
BMI < -2SDS / Feeding difficulties	Yes	Yes	Yes	No	No	NA	1/3	5/5	Yes	Yes	90%	100%	76%	70%	90.9%	100%
motor/cognitive delay	Not yet*	Not yet*	Normal	Yes****	Yes****	NA	1/3	1/5	Yes	Yes	65%	NA	33/15%	NR	NA	NA
NHS	3/6	3/6	5/6	1/6	0/6	NAp	NAp	NAp	3/6	3/6	NAp	NAp	NAp	NAp	NAp	NAp
Further comments				37 genes affected	119 genes affected, BWS features	in abortion with placental mesenchymal dysplasia	Dup of *GRB10, IGFBP1, IGFBP3, (EGFR)*									

*At the age of 2.7 years, **size might be influenced by ethnic origin

***Small but does not fulfil the NHS criterion

****Assumably due to the size of the CNV and its gene content, NA not assessed, NR not reported, NAp not applicable

The *PEG10*:TSS DMR as the second DMR on the long arm of chromosome 7 has not yet been regarded as a candidate gene, as it is not always affected by the aforementioned segmental upd(7q)mat. A common imprinting cluster in *SGCE* has been suggested in pigs (14), a gene which is associated with myoclonus dystonia 11 (OMIM #159,900). In fact, myoclonus dystonia has been documented in single patients with upd(7)mat (15), but it is not a common observation in this cohort (for review: [1]).

The *GRB10*:alt-TSS DMR as the third DMR on chromosome 7 regulates the expression of the growth factor receptor-bound protein 10 (*GRB10*). GRB10 is an adapter protein and member of several signalling pathways (for review: [16]) with a role in cell proliferation, apoptosis and metabolism. In addition to the impact of GRB10 on growth, a negative correlation between *GRB10* expression and head circumference has been suggested [17]. In mice, Grb10 has been shown to contribute to social behaviour [18].

The imprinting signature of *Grb10*/*GRB10* depends on tissue and stage of development and differs between mice and human [19, 20]. In mice, the maternal *Grb10* allele is expressed in nearly all tissues, and inactivation of the maternal allele results in placental and foetal overgrowth [21] whereas disruption of the paternal allele does not affect growth [22]. Furthermore, prenatal overexpression of *Grb10* appears to cause intrauterine growth retardation [23]. In human, *GRB10* is expressed biallelically in the majority of foetal tissues [19]. However, both in mice and human placenta it is expressed from the maternal allele only, suggesting that the maternal *Grb10*/*GRB10* expression in placenta is evolutionarily important for the control of foetal growth [20]. This observation as well as the function of GRB10 as growth inhibitor fits with the intrauterine growth retardation of patients with *GRB10* duplications [24, 25] and upd(7)mat.

## Patients (Table 2)

**Table 2:** Overview on the patients with chromosome 7 disturbances included in this study, and the analysed tissues and conducted tests

Patients	GRB10Del	UPD7M	UPD7qM	GRB10GOM
Molecular disturbance	Deletion in *GRB10*	Maternal UPD of whole chromosome 7	Maternal UPD of chromosome 7qter, LOM of *MEG3*:TSS DMR	GOM of *GRB*10:alt-TSS DMR and 20q13 deletion
Number	2 (twins)	3	1	1
tissue for DNA testing	blood	blood	fibroblasts	blood
DNA tests	(MS-)MLPA CytoScan™ HD Array GS	MS-MLPA	MS-MLPA	MS-MLPA CytoScan™ HD Array GS
Tissue for RNA analysis	blood	fibroblasts	fibroblasts	NA
RNA assays	RNAseq	RNAseq (triplicates) qPCR (triplicates)	RNAseq (triplicates) qPCR (triplicates)	NA

*UPD* uniparental disomy; *LOM* loss of methylation; *GOM* gain of methylation; *GS* genome sequencing.

**Fig. 1:**
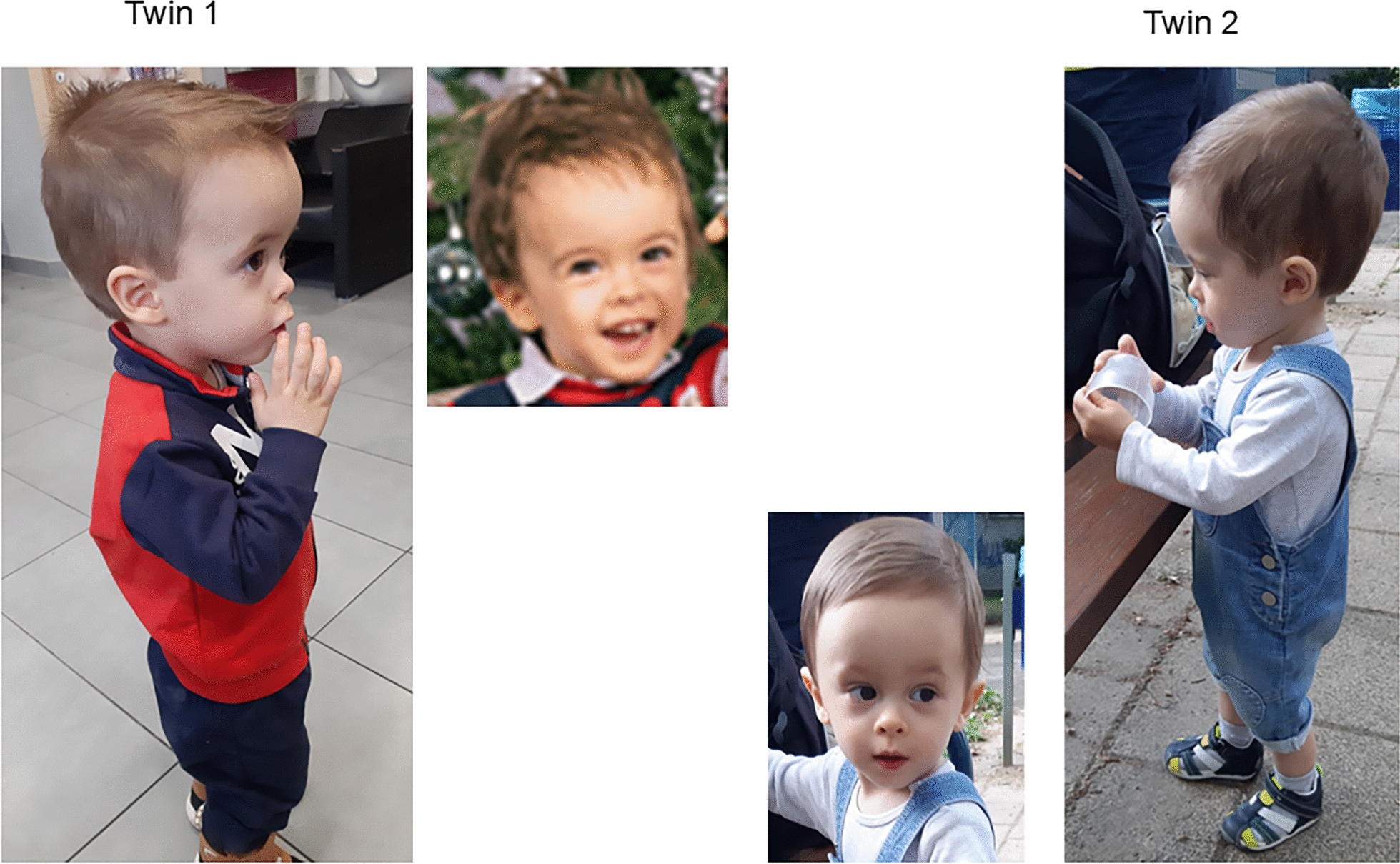
Twin patients with GRB10Del at the age of 2.7 years

**Fig. 2:**
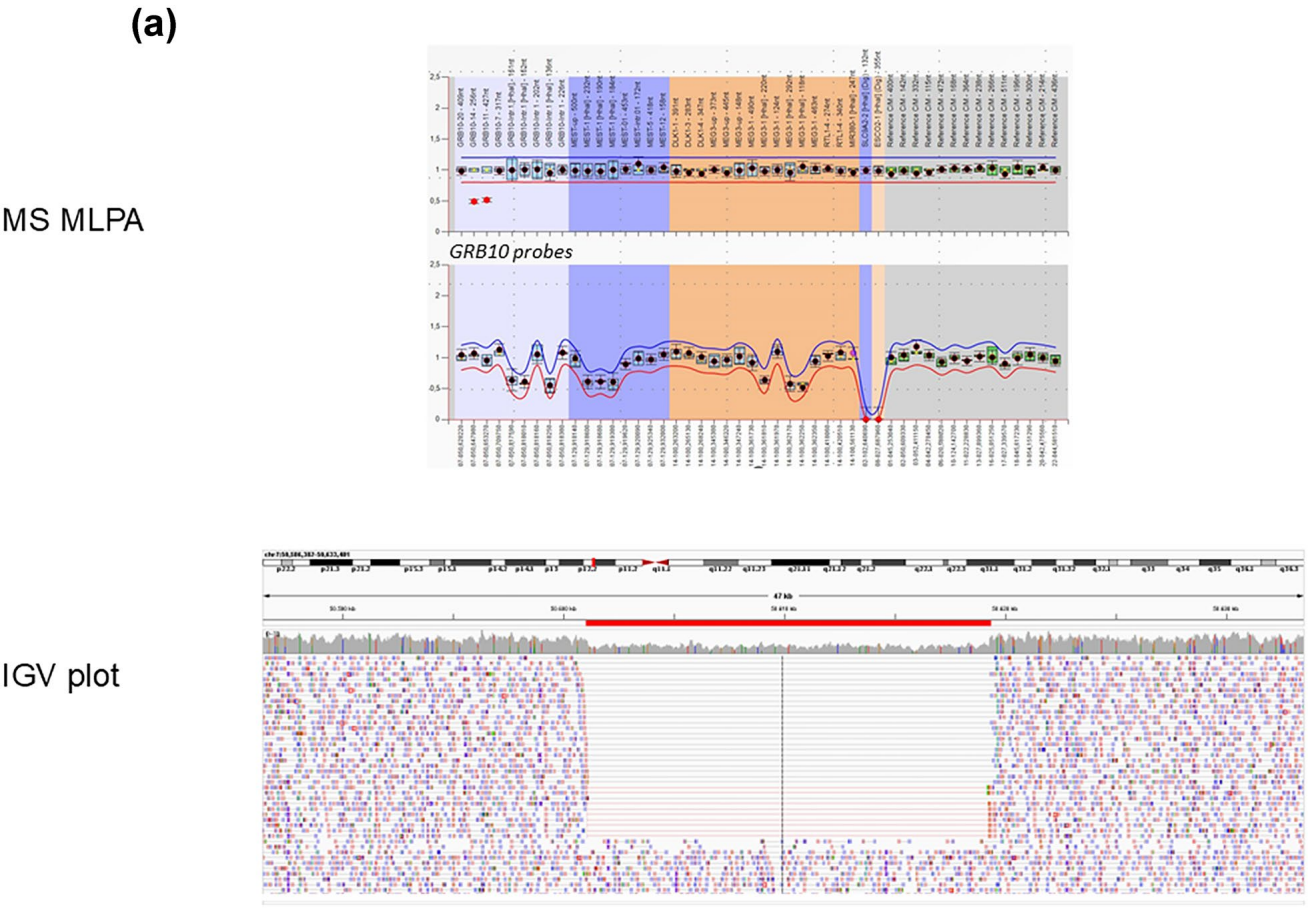

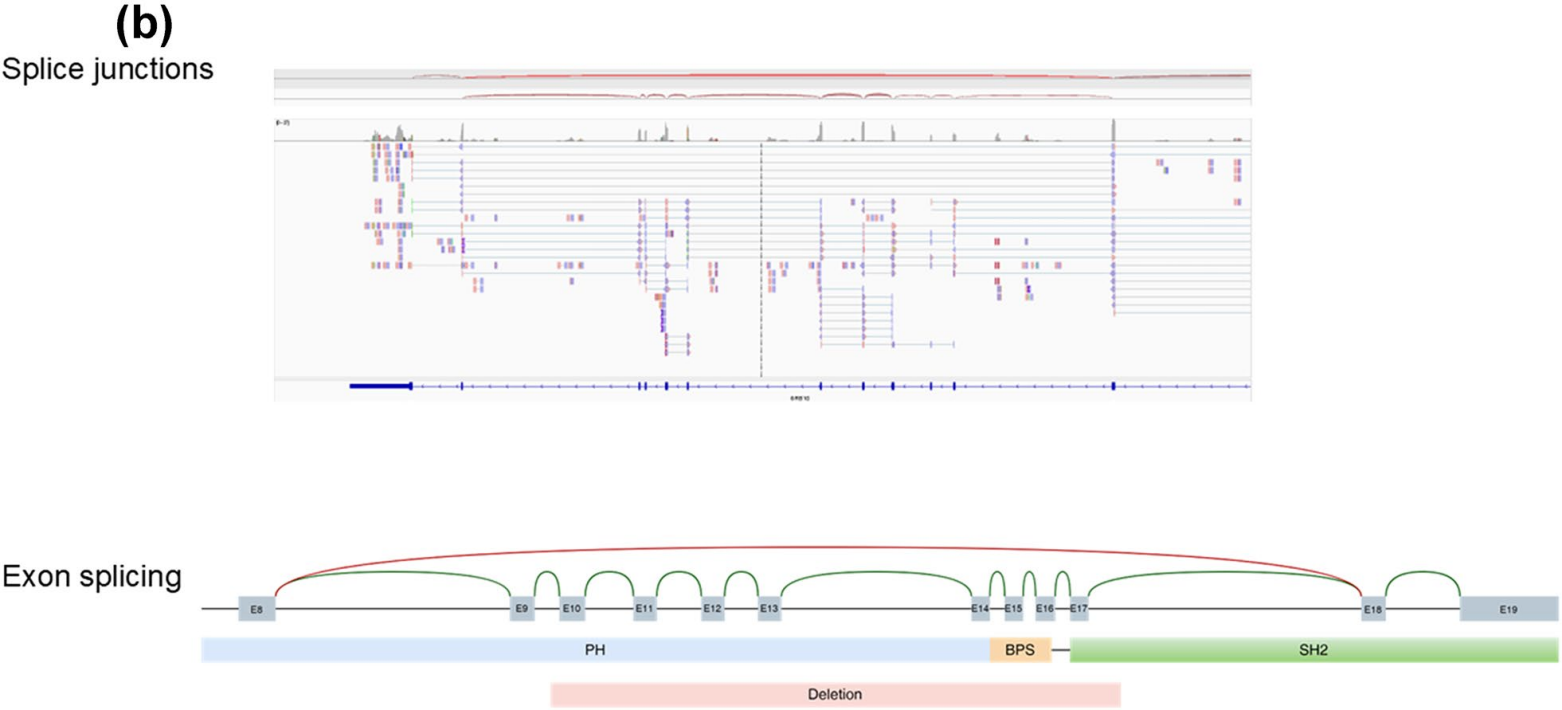
Molecular results of GRB10Del twin 1. **a** DNA results for twin 1: MS-MLPA revealed a deletion of two probes in the CNV run (upper row), whereas the methylation-specific probes were not affected (lower row). IGV (integrative genome viewer) plot of GS data visualizing the 18 kb heterozygous GRB10 deletion. **b** RNA results:—IGV plot of RNA sequencing data showing the read distribution and spanning of splice sites/exon-junctions.—Schematic illustration of the effect of the deletion (red bar) on the splicing of the affected *GRB10* allele delineated from the RNAseq data (green, normal mRNA splicing; red, altered splicing by the deletion). PH, BPS SH2 bars represent the functional domains of GRB10. Notably, exon 9 which is not part of the deletion is also skipped during mRNA processing

The patient group comprised three SRS patients with upd(7)mat (UPD7M), a previously published case with a segmental upd(7q)mat and a *MEG3*:TSS DMR loss of methylation [26](UPD7qM), a twin pair with postnatal SRS features carrying a deletion within *GRB10* (GRB10DEL)(Figs. 1, 2a; Table 2), and a previously reported patient, who is carrier of a gain of methylation of the *GRB*10:alt-TSS DMR and a 20p13 deletion (GRB10GOM) [27].

The study was approved by the ethical committee of the Medical Faculty of the RWTH Aachen (EK303-18, EK159/08).

### Clinical description of the GRB10DEL twins

The monozygotic twin brothers were born as first children to a healthy unrelated Hungarian couple (maternal age: 33 years, paternal age: 39 years). Parental heights were within the normal range but at the lower end (mother 160 cm, z-1.26; father 173 cm, z-1.55). Pregnancy occurred spontaneously and was normal until gestational week 25 (gw) when HELLP syndrome (Haemolysis, Elevat Liver enzymes, Low Platelet) was diagnosed, prompting caesarean section at gw 31.

Twin 1 with a length (43 cm, z 0.36), weight (1740 g, z 0.25) and head circumference (OFC, 30 cm, z 0.26) at birth was in the normal range. At the age of 2.7 years, he was referred to the paediatric hospital due to growth retardation with a height of 88 cm (z-1.51) and a weight of 10 kg (z-2.5). Body mass index (BMI) was 12.9 (z-2.79) (Fig. 1). In contrast, OFC was in the normal range (51 cm (z 0.47)). At that age, the boys showed muscular hypotonia. In addition to his relative macrocephaly, the patient exhibited a protruding forehead, an epicanthus, a broad nasal root, a small mouth and micrognathia. Sitting was achieved at the age of 10 months, walking without help at age of 15 months. Development was documented as normal. Clinical scoring revealed a Netchine–Harbison score (NHS) of 3 out of 6 (relative macrocephaly, protruding forehead, BMI ≤ -2 SD).

Twin 2 showed similar sizes at birth (length: 43 cm, z 0.36; weight 1300 g, z-0.99; OFC 30 cm, z 0.26), but growth retardation was more severe at the age of 2.7 years with a height of 83 cm (z-2.84) and weight of 9.15 (z -3.22), but OFC in the normal range (49.5 cm, z-0.74)(Fig. 1). BMI was 11.8 (z -4.36). At that age, a ventricle septum defect was diagnosed as well as muscular hypotonia. The facial gestalt corresponded to that of his brother, with protruding forehead, a small mouth and micrognathia. NHS was determined as 4 out of 6 (short stature, relative macrocephaly, BMI ≤ -2 SD, protruding forehead). Development was similar to that of his twin brother.

To further compare the clinical findings of the twin pair with upd(7)mat patients, clinical data from the literature and a cohort of 34 patients with SRS features and molecularly confirmed upd(7)mat at the institute in Aachen were evaluated (Table 1).

## Materials and methods

In the patients with UPD7M, UPD7qM and GRB10GOM, the molecular alterations were diagnosed by methylation-specific multiplex ligation-dependent probe amplification (MS-MLPA) tests and microsatellite typing. The GRB10DEL twins were identified by the copy number analysis tool of the MS-MLPA assay.

All genomic analyses in the GRB10DEL carriers and the GRB10GOM patient were conducted on the basis of genomic DNA from peripheral lymphocytes.

RNA analyses in the UPD7M and UPD7qM patients were based on RNA from fibroblasts and compared with data from age-matched controls. In case of the GRB10DEL twins, RNA was isolated from peripheral blood.

### *DNA experiments (*Table 2*)*

Due to the clinical diagnosis of SRS in the twin pair and the molecular heterogeneity of the disease [2], routine diagnostic testing was conducted targeting all clinically relevant differentially methylated regions in 11p15, 7p12.1, 7q32, 6q24, 14q32, 15q11 and 20q13 by methylation-specific multiplex ligation probe-dependent amplification (MS-MLPA; Assays ME030, ME032 and ME034 from MRC Holland, Amsterdam/NL).

The deletion was confirmed by molecular karyotyping using SNP array genotyping (CytoScan™ HD Array, Life Technologies, Carlsbad/USA).

Genomic sequencing (GS) of the DNA samples of the GRB10DEL twins and their parents as well as of the previously published GRB10GOM patient [27] was conducted by using the DNA PCR-free kit (Illumina Inc. San Diego, CA, USA) and sequencing was performed on a NovaSeq 6000 (Illumina Inc.), 2 × 158 cycles. Data analysis was performed with the Illumina DRAGEN-Pipeline (Version: 07.021.645.4.0.3) using hg38 reference genome. Tertiary analysis was performed using both the Emedgene software (Illumina Inc.) and an in-house pipeline. In brief, the in-house pipeline utilizes KGGSeq (v1.2/06/Nov./2022) for variant filtering and annotation. Variants with a minor allele frequency higher than 0.75% in public databases (gnomAD) were discarded. Variant prioritization and evaluation of pathogenicity were based on different prediction tools (CADD, PolyPhen, SIFT, Mutation Taste, Revel, SpliceAI) and variant frequency in public databases. 

Copy number variants (CNV) were analysed by an in-house pipeline, using CNVkit. The detected CNVs were annotated with in-house cohort frequencies and visualized utilizing a CNVizard [28]. Variants were additionally analysed with the Emedgene software (Illumina Inc.) to further assess intronic variants, pathogenic repeat expansions and SVs. 

For the determination of UPDs, the AltAF tool [29] was used which predicts isodisomies via runs of homozygosity on each chromosome and heterodisomies via the inheritance ratio of maternal and paternal SNPs per chromosome [18].

### *RNA experiments (*Table 2*)*

Primary human fibroblasts from the UPD7M and UPD7qM patients, as well as unaffected control samples were derived from skin biopsy samples, were cultured in DMEM with 10% FBS and 5% CO_2_ at 37 °C. Fibroblasts were seeded for 24 h prior to RNA preparation with 1.5 × 10^4^ cells per well on 6-well plates. RNA was extracted with the NucleoSpin RNA Mini kit (Macherey–Nagel) according to the manufactures protocol.

RNA from the two GRB10DEL twins and control samples was isolated from blood taken in S-Monovette® RNA Exact.

RNA concentration and integrity were verified on a TapeStation (Agilent Technologies, St Clara/USA).

Libraries for RNAseq were prepared using either the NEBNext® Ultra™ II Directional RNA Library Prep Kit together with the NEBNext® rRNA Depletion Kit (human/mouse/rat) or QuantSeq FWD V2 kit (Lexogen) according to the manufacturer’s protocol. Sequencing was performed on an Illumina NovaSeq6000 at 2 × 150 bp generating 15–20 million reads per sample. Raw data were demultiplexed, and FASTQ files were generated using bcl_convert. Data were aligned to the GRCh38p4 genome, counted with Star Aligner and further analysed and visualized with BioJupies using default parameters [30]. For differential expression (DE) analysis, three fibroblast cell lines from healthy donors were compared to the patient-derived cell lines and three independent biological replicates (*n* = 3) were performed for each cell line. To calculate DE, we either compared the three controls with each of the patient cell lines individually or by combining the values of all four patient cell lines. Differential expression analysis was performed DESeq2 as provided by the GENAVi environment [31] and iDEP [32]. For the GRB10DEL patients and controls, no biological replicates were available; for the differential expression analysis, the results from the twins were grouped and compared to those of eleven healthy controls. The dataset was not filtered; thereby, weakly expressed genes and genes that are not expressed in the analysed tissue were included in the analysis.

The data were evaluated by using quantitative PCR assays for specific genes, and 3´ mRNAseq of the UPD7M and UPD7qM fibroblasts (suppl. Figure 1).

Two-dimensional principal component analysis (PCA) of differentially expressed genes (DEG) analysis was performed using standard parameters of the GENAVi RNA analysis software package [31].

## Results

### Genomic analysis in the GRB10DEL twins and the GRB10GOM patient

MS-MLPA for the imprinted loci on chromosome 7 in the twin patients revealed a deletion of two probes targeting the *GRB10* gene in 7p12.1 in both twins (Fig. 2). Further copy number variants in other clinically relevant differentially methylated regions were not observed, and MS-MLPA revealed normal methylation patterns for all methylation-specific loci, including those targeting the *GRB10*:alt-TSS DMR.

By trio GS, the exact breakpoints of the deletion could be determined, and the de novo occurrence could be confirmed. Furthermore, the paternal origin of the affected allele could be delineated. The deletion affected a 18 kb region (chr7:50,600,324–50618447)del (hg38))(Fig. 2) including the exons 10 to 17 of the GRB10 gene (NM_001350814.2(GRB10):c.778-308_1544 + 3674del, p.(?)), and thereby affects the functional PH, BPS and SH2 domains. This finding was confirmed by SNP array typing (arr[hg38] 7p12.1(50,603,111–50617998) × 1).

GS did not reveal any other genomic variant related to the clinical features.

Based on the ACMG criteria for pathogenicity classification [33], the available public data and information from the family, the variant was classified as likely pathogenic (PS2, PM2, PM4).

In the previously published GRB10GOM patient [27], GS analysis did not reveal any other pathogenic variant. Furthermore, the re-evaluation of the pathogenicity of the 20p13 deletion indicates that the variant is benign. From these data, it can be delineated that the GOM of the *GRB10*:alt-TSS DMR is functionally associated with the phenotype.

### RNA analysis

RNA sequencing in blood samples of both GRB10DEL twins confirmed the multi-exon deletion within the of the *GRB10* gene (NM_001350814.2) and showed that the functional PH, BPS and SH2 domains were heterozygously deleted (Fig. 2).

**Fig. 3:**
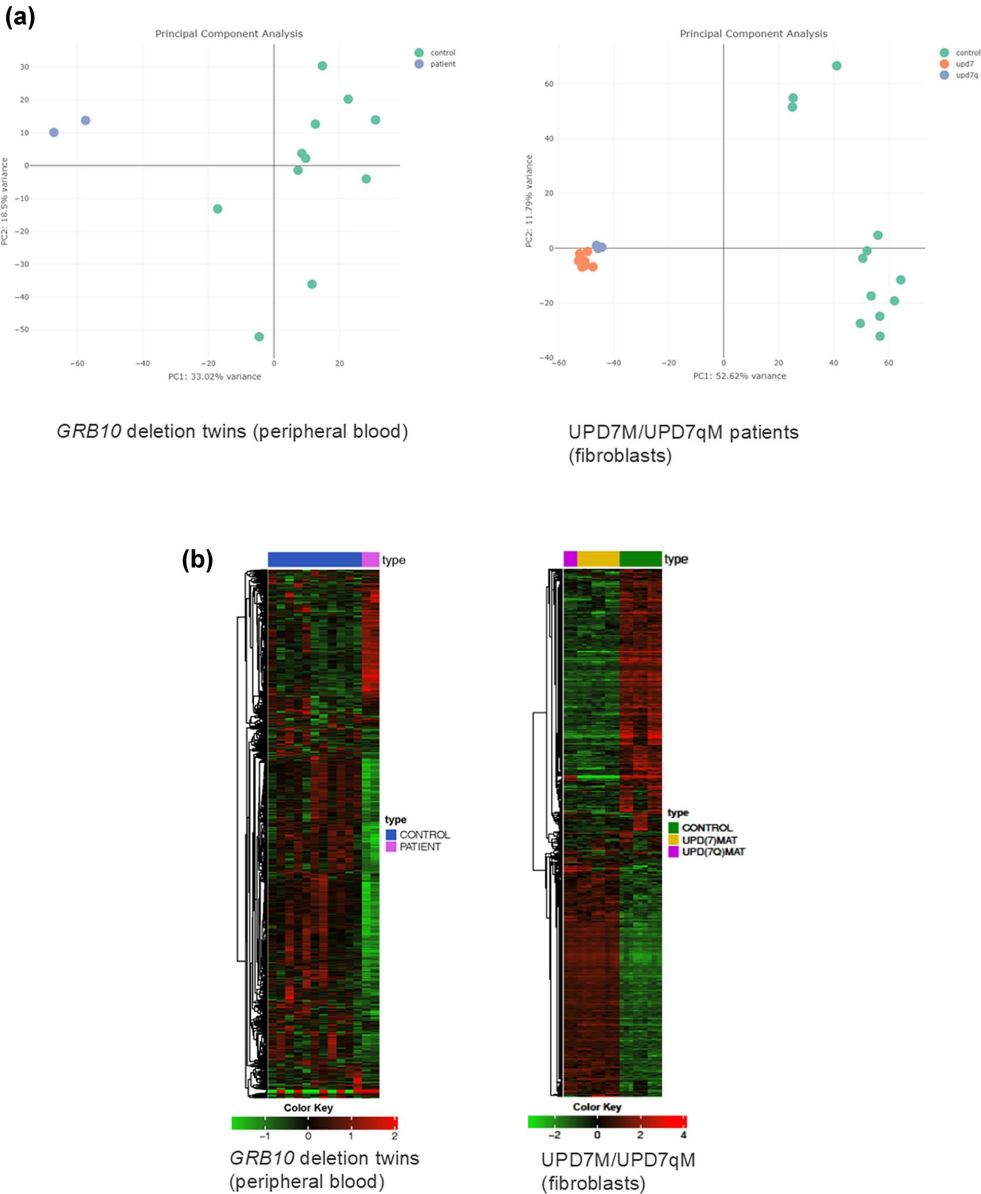

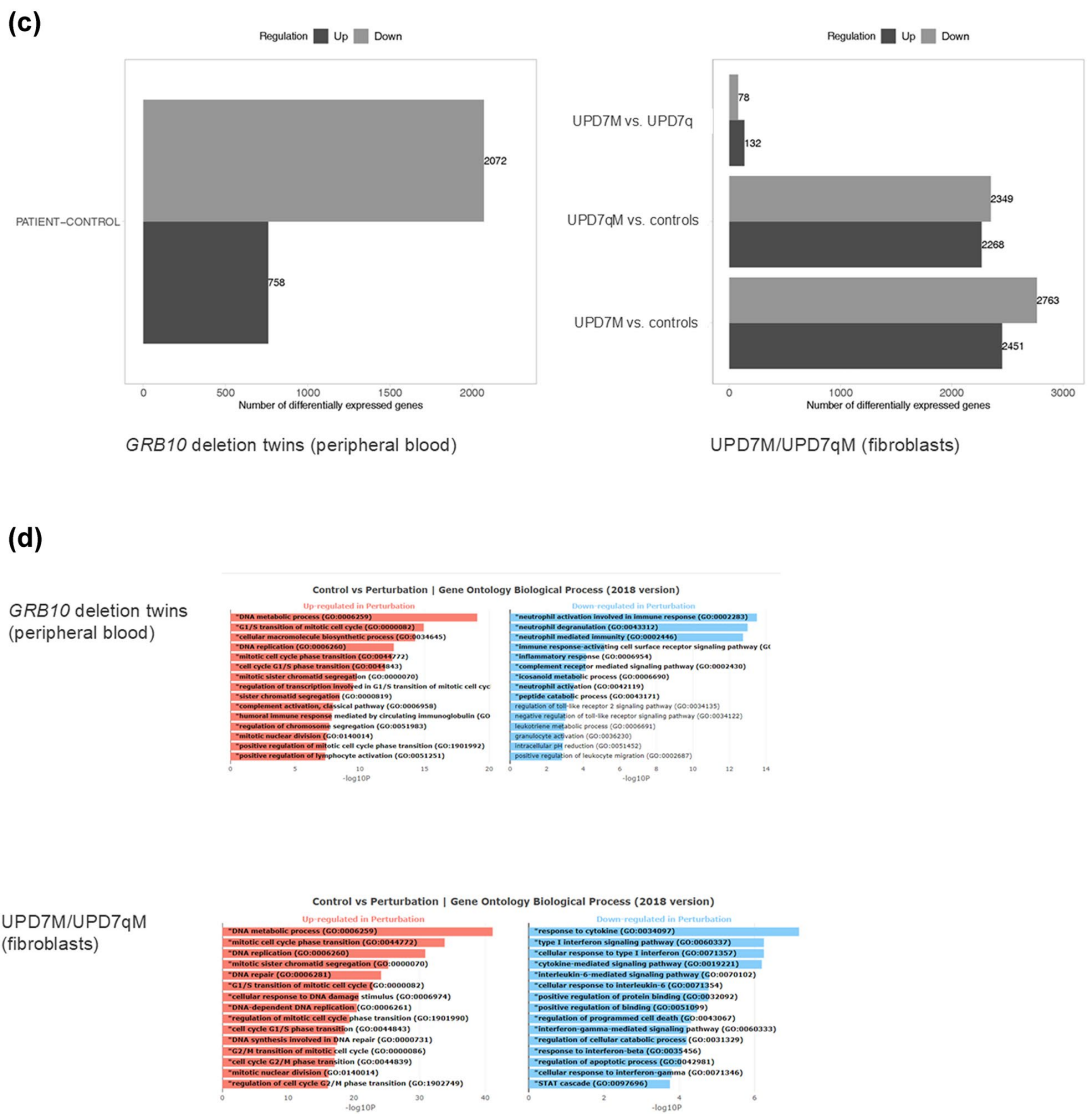
Impact of upd(7)//7q)mat and the deletion in *GRB10* on gene expression. **a** PCA plots of the full-length RNAseq data obtained from the GRB10DEL twins (blood), and the UPD7M/UPD7qM fibroblasts, and of differentially expressed genes (DEGs) compared to controls. **b** Heatmap visualization showing differential gene expression in the GRB10Del (left), and UPD7MAT/UPD7qMAT patients (right). Green indicates reduced expression, red indicates increased expression).**c** Differential gene expression analysis across the three different genetic constitutions, highlighting the number of genes that are either up- or downregulated. **d** Clustering analysis reveals distinct patterns of gene expression in GRB10DEL and UPD7MAT/UPD7qMAT, respectively

RNA sequencing did not indicate an altered expression of *GRB10* and *MEST*, whereas *IGFBP3* appears to be downregulated (Table 3). The expression of *IGF2* and other imprinted genes in 11p15 were not affected, but *MEG3* and *MEG8* in 14q32 were downregulated as well. Two-dimensional PCA of differentially expressed genes (DEG) as well as heatmap visualization showed differences in the expression pattern in peripheral blood between the twin patients and controls (Fig. 3a, b).

**Table 3:** Expression profiles obtained by RNAseq for the twins with GRB10DEL from blood, and from fibroblasts of patients with upd(7)mat and upd(7q)mat/*MEG3*-LOM, respectively

Chromosome	Gene	GRB10 del		upd(7)mat fibroblast vs control		upd(7q)mat /*MEG3* DMR hypo—fibroblast vs control		Imprinting status*	Expressed allele
		PATIENT-CONTROL_log2FC	PATIENT-CONTROL_adjPval	UPD(7)MAT-CONTROL_log2FC	UPD(7)MAT-CONTROL_adjPval	UPD(7Q)MAT-CONTROL_log2FC	UPD(7Q)MAT-CONTROL_adjPval		
2q35	*IGFBP2*	− 1,905216719	1	− 1,298383931	1	0,564102746	1	Not imprinted	Biallelic
2q35	*IGFBP5*	3,054635309	1	− **6,1523522**	3,19E-45	− **3,360727724**	6,39E-05	Not imprinted	Biallelic
2q35	*IGFBP6*	− 1,059585104	1	− 0,986294443	1	− 1,443158144	1	Not imprinted	Biallelic
3q27.2	*IGF2BP2*	− 1,529679293	1	− 0,377414176	1	− 0,264653497	1	Not imprinted	Biallelic
4q12	*IGFBP7*	− 0,646687108	1	− 1,283546799	7,35E-01	− 1,155871638	1	Not imprinted	Biallelic
6q24.2	*PLAGL1*	− 1,312633621	1,00E + 00	0,048701967	1	− 0,212234123	1	Imprinted	Paternal
6q25.3	*IGF2R*	− 1,588867555	5,97E-02	− 1,066350655	1,00E + 00	− 1,096152485	1	Not imprinted	Biallelic
7p12.1	*GRB10*	− 0,62783322	1	0,244434796	1	0,281179968	1	Imprinted	Isoform dependent, biallic in blood
7p12.3	*IGFBP1*	NA	NA	3,380779391	0,066273421	**4,644022158**	0,005646705	Not imprinted	Biallelic
7p12.3	*IGFBP3*	− **4,933152476**	1,85E-05	0,215981085	1	0,380355195	1	Not imprinted	Biallelic
7p15.3	*IGF2BP3*	0,444785162	1	**2,340578399**	4,18E-09	**2,129795736**	0,002131692	Not imprinted	Biallelic
7q21.3	*PEG10*	− 0,276102405	1	− **3,371318339**	1,64E-22	1,713255094	0,107179435	Imprinted	Paternal
7q32	*MEST*	0,205892215	1	− **6,605327276**	8,16E-97	− **6,176855751**	6,31E-39	Imprinted	Paternal
8q12.1	*PLAG1*	0,257923332	1	− 0,763615277	1,00E + 00	− 0,643518551	1	Not imprinted	Biallelic
11p15.5	*CDKN1C*	− 0,013958553	1	− 4,832183999	1,41E-13	− **3,255119267**	0,000238746	Imprinted	Maternal
11p15.5	*H19*	0,257213154	1	− **2,825088113**	2,22E-02	− 2,324791455	0,804842853	Imprinted	Maternal
11p15.5	*IGF2*	− 0,431723244	1	− 3,308172384	0,123639369	− 3,315291364	NA	Imprinted	Paternal
11p15.5	KCNQ1	− 0,3610377	1	1,356393782	1	− 0,430584995	NA	Imprinted	Maternal
11p15.5	*KCNQ1OT1*	− 0,269774594	1	− 0,522379913	1	− 0,347537554	1	Imprinted	Paternal
14q32	*MEG3*	− **7,77810641**	0,0218278	0,345334342	1,00E + 00	1,822036995	7,57E-07	Imprinted	Maternal
14q32	*MEG8*	− 2,973101519	1	0,557669297	1,00E + 00	2,017753361	2,04E-08	Imprinted	Maternal
17q21.2	*IGFBP4*	1,576787869	1	− 1,220180311	8,43E-01	− 1,261527562	1	Not imprinted	Biallelic
17q21.32	*IGF2BP1*	NA	NA	1,351181533	5,59E-01	1,796772486	0,058690002	Not imprinted	Biallelic

Only data for selected genes regarded as SRS candidate genes or interacting with them are shown. (Log2FC; adjPval adjusted p-values for differential expression; NA not assessed; Green: > twofold upregulated gene expression; red, < twofold downregulated gene expression.

Bold statically significant *p* < = 0.05; Background colour groups genes per chromosome;

*Imprinting and expression according to https://www.geneimprint.com/site/genes-by-species).

Using two-dimensional PCA, we were able to visualize the clustering of two groups, corresponding to our group labels (control and patient). In general, the control group shows a broader variance on the y-axis (PC2), probably due to the age difference in the control group. The variance in the control group is even higher in the RNAseq data of the GRB10DEL patients. The most reasonable explanation is the high diversity in the control group that consisted of parental samples of mixed age and gender.

More than 2800 genes could be identified to be differentially expressed between the two group: 2072 were down- and 758 were upregulated (Fig. 3c). Gene set enrichment analysis revealed that upregulated genes are involved in DNA metabolism and mitotic cell cycle transition, whereas pathway regulating immune response was downregulated (Fig. 3d).

RNA sequencing in fibroblasts from the three UPD7M and the UPD7qM patients [26] revealed a reduced *MEST* expression in all samples (Table 3), compared to the healthy controls. However, the expression of *GRB10* was normal. The observations for both genes were confirmed by qPCR experiments (suppl. Figure 1). As expected, *PEG10* transcription was only affected in the whole chromosome 7 UPD cases (UPD7M), but not in the fibroblasts from the patient with segmental upd(7q)mat (UPD7qM).

In the UPD7qM patient with additional LOM of the *MEG3*:TSS DMR, *MEG3* and *MEG8* (14q32) were overexpressed, which was not observed for the other UPD7M patients.

The chromosome 11p15 encoded and imprinted genes *IGF2, H19* and *CDKN1C* showed downregulation in both UPD7M and UPD7qM fibroblasts (Table 3).

Among the nonimprinted genes showing an altered expression was observed for *IGFBP1* in 7p12.3, *IGF2BP3* in 7p15.3 and *IGFBP5* in 2q35 (Table 3). RNAseq data did not show an impact of upd(7)mat on *IGFBP3* transcription expression of which was altered in the GRB10DEL twins.

Differential expression between controls and the UPD7M/UPD7qM patients was observed in more than 5,200 genes in UPD7M and 4,600 genes in UPD7qM (Fig. 3). Differences in expression between UPD7M and UPD7qM were detectable only in ~ 200 genes. Corresponding to the observation in the GRB10DEL twins, gene set enrichment analysis revealed that genes with a role in DNA metabolism and mitotic cell cycle transition were upregulated, whereas immune response was downregulated (data not shown).

## Discussion

Though upd(7)mat was the first consistent molecular alteration identified in patients with SRS features [4], the search for the disease-causing gene(s) on chromosome 7 is still ongoing.

We therefore carried out expression studies in tissues from SRS patients with chromosome 7 disturbances, and thereby aimed to decipher the contribution of this chromosome to the aetiology of SRS features.

In general, analysis in respect to differential gene expression between patients and controls, RNAseq data from UPD7M, UPD7qM and control fibroblasts exhibited significant differences in gene expression, and gene ontology annotations showed altered expression of genes regulating DNA metabolism and mitosis and thereby factors that promote cell and tissue growth (Fig. 3).

In the last years, numerous case reports have indicated that the chromosomal region 7q32 harbours a SRS causing gene, and based on CNVs in that region and segmental upd(7q)mat, there is growing evidence that *MEST* is involved in the pathology of SRS (Table 1). This assumption is now corroborated by the observation that its expression is significantly reduced in patients with upd(7)mat and upd(7q)mat, respectively (Table 3). As carriers of upd(7)mat and patients with 7q32 disturbances often exhibit the full clinical spectrum of SRS, it can be delineated that haploinsufficiency of *MEST* caused by upd(7)mat affects functional pathways associated with the disease, including factors contributing to DNA metabolism, regulation of mitosis and immune response. In contrast, overexpression of *MEST* which can be expected in case of upd(7)pat does not cause a uniform phenotype. Thus, haploinsufficiency of *MEST* is probably of functional relevance. However, with the exception of a role in fat mass deposition and behaviour [13], the biological function of *MEST* still remains unknown.

Transcription of *PEG10* as the second imprinted gene on chromosome 7 was reduced as well in UPD7M patients, but as expected it was not altered in the fibroblast from the UPD7qM patient (Table 3). Accordingly, a physiological role in the aetiology of SRS is currently not obvious.

*GRB10* (7p12.1) transcription was in the normal range in the UPD7M and UPD7qM/*MEG3*-LOM fibroblasts (Table 3); therefore, the contribution of *GRB10* via a upd(7)mat mediated mechanism at least for the postnatal phenotype of SRS is questionable.

Whereas expression data from upd(7)mat cells have not yet been reported in the literature, comparable data are available for SRS patients with IC1 LOM and TS14 patients with *MEG3* LOM [34]. Our data from the UPD7qM/*MEG3* LOM fibroblasts correspond to that of the TS14 patients in respect to *MEG3* and *MEG8* upregulation (Table 3). In the study from Abi Habib et al. [34], *MEST* was not analysed, but for *GRB10* the expression was comparable to that of controls, as is in the UPD7M and UPD7qM fibroblasts in this study.

The impact of upd(7)mat on the expression of *IGFBP1*, *IGF2BP3* and *IGFBP5* needs further confirmation, and their putative contribution to SRS features by disturbances of the IGF1 system or independent pathways needs further research (for review: [35]).

Interestingly, the expression of *IGF2* was downregulated in fibroblasts from upd(7)mat patients and in the upd(7q)mat/*MEG3* LOM cells, corresponding to the effect expected for IC1 LOM as the major molecular alteration in SRS. This observation corresponds to the findings in IC1 LOM and TS14 fibroblasts [34] and suggests that *IGF2* expression is influenced by the altered expression of factors encoded by chromosome 7 as well as by overexpression of *MEG3* and *MEG8*, as suggested by Abib Habib et al. [34].

Unexpectedly, in UPD7M and UPD7qM fibroblasts downregulation of *H19* regulated by the IC1 in 11p15.5, and *CDKN1C* located in the IC2 in 11p15.5 was observed. In fact, these findings are at first glance contradictory to the expected upregulation of *H19* in IC1 LOM patients, and to the function of *CDKN1C* as a growth inhibitor as well the reports on pathogenic gain-of-function *CDKN1C* variants in SRS patients (for review: [36]). However, it should be emphasized that the physiological role of H19 and its alternative transcripts is mainly unknown, and that *CDKN1C* shows a specific temporal and spatial expression. Therefore, it remains questionable whether the observed dysregulation of these genes is of functional relevance.

Whereas the pathoetiological role of *GRB10* has remained unclear from the upd(7/7q)mat fibroblasts studies, further insights into its contribution to the SRS phenotype could be obtained from the twin pair with an intragenic *GRB10* deletion and postnatal manifestation of SRS features.

The heterozygous de novo deletion of the paternal allele in the twins affects exons 10–17 of the *GRB10* gene and results in an aberrant transcript lacking exons 9–17. On protein level, three out of five protein binding regions are affected which are essential for GRB10 function (for review: [16]; Fig. 2).

The physiological relevance of the increase of *IGFBP3* expression currently remains unclear and needs further studies.

The lack of IUGR as a major clinical feature of SRS in the GRB10DEL twins on the paternal allele is in accordance with the observation that its homologous gene in mice is a growth suppressor [23], which is expressed from the maternal allele only in the placenta [20]. Duplications of the maternal *GRB10* copies should therefore result in IUGR, whereas the paternal *GRB10* allele is silenced and does not alter prenatal growth (Table 1) [37, 38]. Thus, overexpression of the maternally transmitted *GRB10* copy probably contributes to prenatal growth restriction in chromosome 7-linked SRS, but it should be noted that disturbance of the *MEST* region in 7q32 alone is sufficient to cause IUGR in SRS (Table 1).

The postnatal contribution of *GRB10* to the SRS phenotype is rather difficult to estimate, due to the at first glance heterogeneous phenotypes in patients with chromosome 7p12 disturbances. However, the inclusion of clinical features in the delineation of functional consequences of these alterations has to consider the general clinical heterogeneity of SRS even in patients with the same molecular defect (Table 1). In fact, the GRB10DEL twins in this study present the full postnatal clinical picture of SRS, including PNGR and relative macrocephaly with the typical facial gestalt (Fig. 1). It can therefore be concluded that the three functional *GRB10* domains on the paternal allele affected in the GRB10DEL twins play a role in growth pathways and that their absents contributes to the postnatal SRS phenotype.

PNGR is a typical feature for all phenotypes associated with chromosome 7 disturbances listed in Table 1 with the exception of the two patients with larger 7p12 deletions [38, 39]. In these two cases, more than 30 genes are affected, and therefore, a genotype–phenotype correlation is difficult. The molecular patterns of the other cases reveal a consistent pattern resulting in the suppression of the paternal *GRB10* allele (upd(7)mat, deletion of *GRB10)* or functional gain of the maternal *GRB10* allele (duplication, GOM). The different physiological pathways to which *GRB10* probably contributes might explain the functional consequences of silencing the paternal allele or overexpression of the maternal allele. In fact, the best known mechanism of GRB10 function is its growth inhibitory effect via insulin signalling by interacting with the insulin receptor and inhibition of the downstream PIK3K/AKT and MAPK pathways. This inhibition has a negative impact on metabolic synthesis and storage, as well as on cellular growth (for review: [16]). As described for IUGR, PNGR in case of *GRB10* duplications might also be explained by an increase of GRB10 dosage [23], but due to its biallelic expression in many human tissues this explanation might not be apply for the molecular disturbances which silence the paternal allele (upd(7)mat, deletion or GOM of *GRB10)*. These defects should cause disturbances of other growth pathway in which GRB10 might additionally be involved [23].

Duplication of the maternal *GRB10* allele in combination with an unaffected paternal allele does not seem to cause relative macrocephaly [24, 25], whereas disturbance of the paternal allele, either by deletion (twins in this study) or maternalization of the paternal allele in the GRB10GOM patient [27] results in an increased OFC. As the latter (GOM of *GRB10*) is functionally comparable to upd(7)mat, relative macrocephaly in upd(7)mat appears to be caused by the lack or disturbance of the paternal *GRB10* allele. The mechanism behind these observations might consist of an alternative transcript which is monoallelically expressed from the paternal allele, but which has not yet been identified but conceivable due to the complex isoform- and tissue-specific expression of *GRB10* [19]. But again, pathogenic variations of the imprinted 7q32 region are associated with the SRS phenotype as well, as described for IUGR.

## Conclusions

In summary, the clinical observations in patients with different 7p12 disturbances and upd(7)mat patients (Table 1) as well as the RNAseq results show that reduced or perturbed expression of the paternally inherited alleles of both *MEST* in 7q32 and *GRB10* in 7p12.1 are associated with similar SRS features.

Though it should be emphasized that the results from UPD7M/UPD7qM and *GRB10DEL* patients were achieved from different tissues and are based on a small number of samples, our data allow to delineate their contribution to the SRS phenotype:

- IUGR as the prenatal phenotype of upd(7)mat is caused by defective paternal alleles of the 7q32 region, as well as by overexpression of the maternal *GRB10* allele [23] whereas defective *GRB10* paternal alleles do not cause this feature. Disturbances of both regions can cause IUGR synergistically, as well as separately.
- The altered expression of *MEST* in 7q32 by upd(7)mat is associated with the complete SRS phenotype, but the functional link is currently unclear.
- Silencing or maternalization of the paternal *GRB10* copy and duplication of the chromosomal region 7p12 are associated with a postnatal SRS-like phenotype.
- Learning difficulty and cognitive impairment of upd(7)mat patients are rather not associated with *GRB10* as the *GRB10DEL* twins show a normal development, but an impact on social behaviour cannot be excluded [18].
- Functionally, the disturbance of chromosome 7(q) encoded genes causes dysregulation of genes in the SRS-associated 11p15.5 region and factors involved in the function of IGF2 as one of the SRS genes. These findings further corroborate their physiological interaction and/or their synergistic interaction in the imprinted gene network [40].

## Supplementary Information

Additional file1 Supplementary Figure 1: Results from qPCR studies in fibroblasts from the UPD7M and UPD7qM patients for different genes localised in imprinted regions on chromosomes 6, 7, 11 and 14. Quantitative Real-Time PCR (qRT-PCR) was performed using Platinum qPCR SuperMix UDG (Invitrogen, Carlsbad/CA, USA), TaqMan® Gene Expression Master Mix (LifeTechnologies, Darmstadt, Germany) according to standard protocols. qRT-PCR reactions were run in 10 μl reactions, using the standard reaction protocol. Further details are available on request. The qRT-PCR runs were performed on a StepOnePlus with the StepOneTM-Software v.2.2.1 (Applied Biosystems) and the results were calculated with the ΔΔCT method. The reference gene (TBP) and the target genes were always measured in the same qRT-PCR run. The individual experiments were repeated in three biological replicates using different RNA isolates. The control group consisted of fibroblasts derived from healthy individuals of mixed age (7y - >40y) and gender. The individual experiments were repeated in three biological replicates using different RNA isolates. (PDF 131 KB)

## Data Availability

No datasets were generated or analysed during the current study.

## Abbreviations

DMR: Differentially methylated region
GOM: Gain of methylation
IC1, IC2: Imprinting centre 1, 2 (in 11p15.5)
ImpDis: Imprinting disorder
IUGR: Intrauterine growth restriction
LOM: Loss of methylation
MS MLPA: Methylation-specific multiplex ligation-dependent probe amplification
OFC: Occipitofrontal circumference
PNGR: Postnatal growth restriction
SD: Standard deviation
SRS: Silver–Russell syndrome
UPD: Uniparental disomy
Upd(7)mat: Maternal UPD of chromosome 7
Upd(7q)mat: Maternal UPD of the long arm of chromosome 7
