# Supplementary figures and images for: Maternal uniparental disomy of chromosome 7: how chromosome 7-encoded imprinted genes contribute to the Silver–Russell phenotype
Source: Clin Epigenetics. 2025 Apr 30;17:70. doi: 10.1186/s13148-025-01867-3 (PMC12042466; doi:10.1186/s13148-025-01867-3)

# Suppl. Fig. 1

Chromosome 7

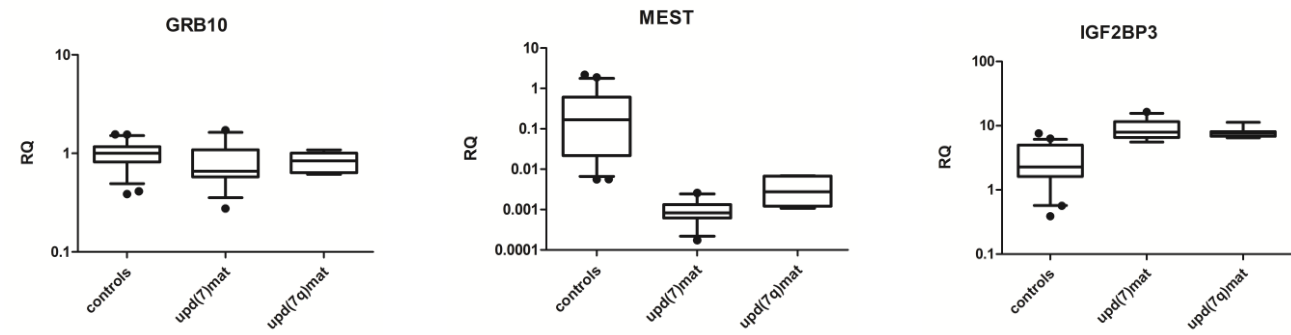

Chromosome 11

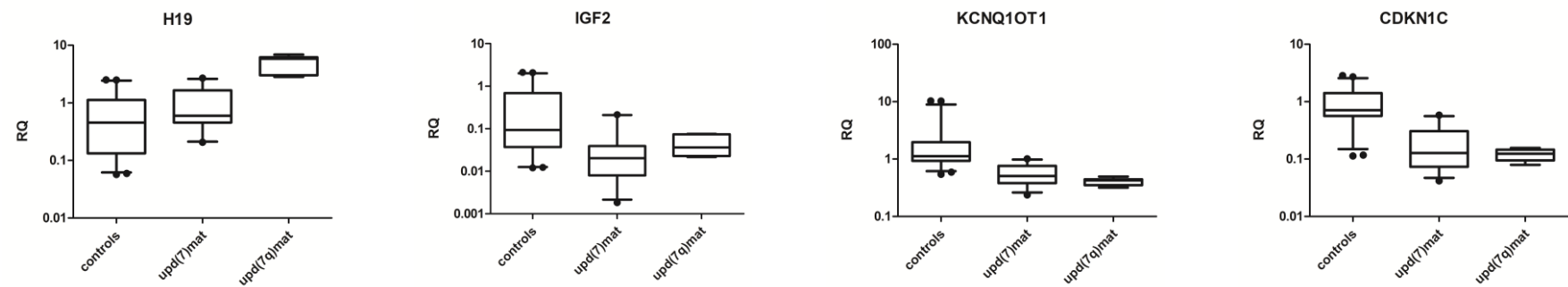

Chromosome 6

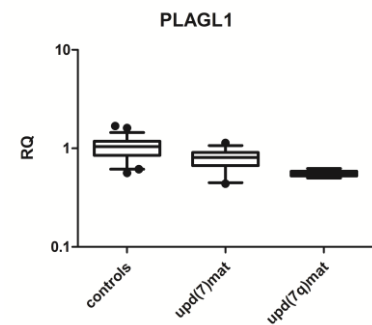

Chromosome 14

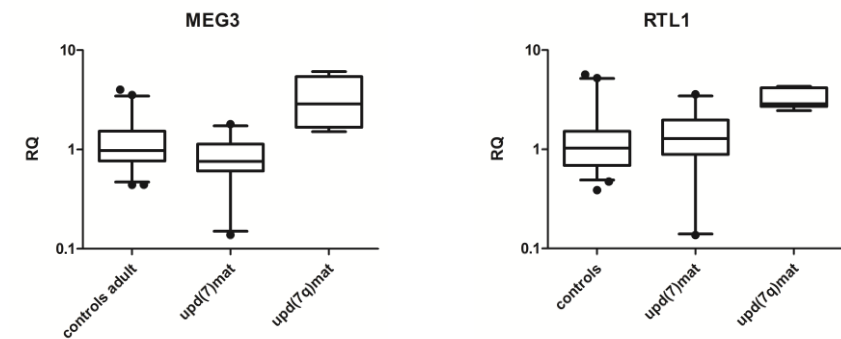

Supplement: Supplementary file 1 — Additional file1 Supplementary Figure 1: Results from qPCR studies in fibroblasts from the UPD7M and UPD7qM patients for different genes localised in imprinted regions on chromosomes 6, 7, 11 and 14. Quantitative Real-Time PCR (qRT-PCR) was performed using Platinum qPCR SuperMix UDG (Invitrogen, Carlsbad/CA, USA), TaqMan® Gene Expression Master Mix (LifeTechnologies, Darmstadt, Germany) according to standard protocols. qRT-PCR reactions were run in 10 μl reactions, using the standard reaction protocol. Further details are available on request. The qRT-PCR runs were performed on a StepOnePlus with the StepOneTM-Software v.2.2.1 (Applied Biosystems) and the results were calculated with the ΔΔCT method. The reference gene (TBP) and the target genes were always measured in the same qRT-PCR run. The individual experiments were repeated in three biological replicates using different RNA isolates. The control group consisted of fibroblasts derived from healthy individuals of mixed age (7y - >40y) and gender. The individual experiments were repeated in three biological replicates using different RNA isolates. (PDF 131 KB) [file 13148_2025_1867_MOESM1_ESM.pdf]
